# Structural divergence and molecular adaptation of *Rhodiola juparensis* organelle genomes

**DOI:** 10.3389/fpls.2026.1881546

**Published:** 2026-07-08

**Authors:** Guocui Deng, Yebing Yin, Kaili Duan, Junze Zhang, Niyang Xiang, Tao Yuan

**Affiliations:** 1 College of Life Science and Technology, Xinjiang University, and Xinjiang Key Laboratory of Biological Resources and Genetic Engineering, Ürümqi, China; 2 State Key Laboratory of Hybrid Rice, Laboratory of Plant Systematics and Evolutionary Biology, College of Life Sciences, Wuhan University, Wuhan, China; 3 School of Ecology and Environment, Tibet University, Lhasa, China; 4 Hubei Key Laboratory of Regional Development and Environmental Response, School of Resources and Environmental Science, Hubei University, Wuhan, China

**Keywords:** adaptive evolution, organelle genomes, positive selection, Qinghai-Tibet Plateau, *Rhodiola juparensis*

## Abstract

**Introduction:**

*Rhodiola juparensis*, a cushion plant, plays a critical role in sustaining the fragile ecological environment of the Qinghai‑Tibet Plateau. Understanding its adaptive evolution is essential, yet the organelle genomic architecture and evolutionary dynamics of this species remain poorly characterized.

**Methods:**

We sequenced and assembled the chloroplast (plastome) and mitochondrial (mitogenome) genomes of *R. juparensis*. Comprehensive comparative analyses were performed on organellar genome structure, RNA editing events, and positively selected genes. Divergence time estimation and genome‑wide positive selection scans were conducted using phylogenetic and bioinformatic approaches.

**Results:**

The plastome exhibited a more conserved structure but a faster nucleotide substitution rate, whereas the mitogenome showed greater structural complexity but a slower substitution rate. Divergence time estimation suggested that rapid differentiation of Crassulaceae species occurred around 7.15 Mya, likely linked to decreasing atmospheric CO₂ levels and the dramatic uplift of the Qinghai‑Tibet Plateau during the Late Miocene. Positive selection analysis identified three candidate genes (matR, ccmFc, and ATP8) under positive selection. Additionally, numerous pentatricopeptide repeat (PPR) proteins were detected in the nuclear genome.

**Discussion:**

The contrasting evolutionary patterns between the two organellar genomes highlight distinct selective constraints and mutation rates. The identified positively selected genes may contribute to mitochondrial function and stress adaptation. The abundance of nuclear‑encoded PPR proteins suggests a potential repair mechanism via RNA editing that could mitigate UV‑induced DNA damage, offering an adaptive advantage in the high‑altitude environment. Collectively, these findings provide valuable insights into the adaptive evolution of *R. juparensis* and the evolutionary history of Crassulaceae on the Qinghai‑Tibet Plateau.

## Introduction

Higher plants possess organelle genomes, including the plastome and mitogenome, which are controlled by the nuclear genome but have their own genetic systems. Hence, these are referred to as semi−autonomous organelles. The mitochondrion and plastid serve as the sites of aerobic respiration and photosynthesis in the cell, respectively, and are essential for proper cellular function. Although the plastome and mitogenome share many characteristics, such as replication (Maréchal and Brisson, 2010) and inheritance patterns (Birky, 1995), there are also notable differences between them. For example, the plastome typically has a circular structure with a conserved quadripartite organization, and is conserved in gene content, gene order, and genome size. In contrast, the mitogenome displays greater variation in structure, gene number, and gene order. A variety of structures exist in the mitogenome, including circular, polycircular, and linear forms (Shen et al., 2019; Zhang et al., 2024, 2025a, 2025b). The size of the plastome in higher plants ranges from approximately 100 to 200 kb (Daniell et al., 2016), whereas the mitogenome size ranges from 186 kb to 1 Mb. The number of genes in the plastome is approximately 120, whereas the number of mitogenome genes ranges from 19 to 50 (Skippington et al., 2015; Johnston and Williams, 2016; Mower, 2020). Nevertheless, precise and stringent regulatory mechanisms exist between mitochondria and plastids. Organelle genomes possess several advantageous properties, including structural simplicity, lack of recombination, and uniparental inheritance. These features have been widely used in studies on phylogeny (Yu et al., 2020), phylogeography (Shaw et al., 2014), and environmental adaptation (Jiang et al., 2018; Chen et al., 2025) across different taxonomic orders. In recent years, the volume of organelle genomic data available, particularly for plastomes, has increased substantially, making it possible to use organelle genomes as a resource for studying adaptive evolution. Although many current studies assume that sequence variation in plastome is selectively neutral, driven by genetic drift (Shaw et al., 2014), a growing body of evidence suggests that positive selection may have played an important role in the adaptive evolution of plastomes (Yu et al., 2023; Lu et al., 2025; Xiang et al., 2026). In particular, many recent studies have identified signatures of positive selection in the plastome sequences of various plant species using comparative genomic approaches, suggesting that the plastome may undergo adaptive evolution in response to environmental changes (Jiang et al., 2018; Zhao et al., 2020). Nevertheless, due to the complexity of plant mitogenomes, few studies have investigated adaptation to extreme environments at the mitogenome level, highlighting the need for the present study on mitogenomes.

*Rhodiola juparensis* belongs to the family Crassulaceae. It is a perennial dwarf herb characterized by short stems, small leaves, and high branch density. It is predominantly found in the subpolar regions of the Northern Hemisphere, especially on the Qinghai−Tibet Plateau (QTP). This plant has evolved a low and dense cushion structure to withstand the extreme environmental conditions of the QTP, such as strong winds and low temperatures (Körner, 2003). This cushion structure results in highly specialized leaves that are either lignified or keratinized, which help reduce evaporation and prevent desiccation. Additionally, the plant has a robust root system, allowing it to absorb water and nutrients and adapt to the nutrient−poor soils of the QTP (He et al., 2007). Cushion plants such as *R. juparensis* serve as “micro−refugia” for species with low stress tolerance in harsh environments (Butterfield et al., 2013). A positive correlation has been observed between the abundance of the cushion plant *Arenaria polytrichoides* and that of other plants at high elevations in the Himalayas of China (Yang et al., 2010). In harsh environments, cushion plants often act as facilitators, nurse plants, and foundation species, making them crucial for maintaining fragile highland ecosystems. Thus, *R. juparensis* serves as a valuable biological resource for studying cushion plant adaptation to the QTP. Although a recent study identified high-altitude acclimation signatures on *Rhodiola* plastomes (Zhao et al., 2020), systematic research on the adaptation of *R. juparensis* plastomes to the QTP is still lacking. Importantly, it remains unclear whether the acclimation imprints observed on plastomes also exist on the mitogenome, and what role the mitogenome plays in environmental adaptation.

Studying adaptation mechanisms in cushion plants such as *R. juparensis* is crucial for understanding how plant species survive in harsh environments and how ecosystems are sustained. Plastids and mitochondria play a pivotal role in photosynthesis, energy metabolism, and respiration, and may also contribute to plant adaptation to extreme environments (Xu et al., 2011; Sultan et al., 2016; Zhao et al., 2020). Therefore, analyzing the mitogenome of *R. juparensis* can provide valuable insights into the adaptation mechanisms of cushion plants on the QTP. In this study, we conducted a systematic comparative genomic analysis of the plastome and mitogenome of *R. juparensis* using high-throughput sequencing. We examined genome structure, gene content, transposable element (TE) types, intracellular gene transfer, and nucleotide substitution rates of protein-coding genes. Furthermore, we identified potential adaptive signatures in the mitogenomes of *Rhodiola* species based on positive selection analysis. Our findings provide a foundation for future studies on the adaptation of *Rhodiola* species to extreme environments.

## Materials and methods

### Plant material

Leaf tissue samples of *R. juparensis* were collected at an altitude of 4,892 m (92.36°E, 29.78°N) on the QTP. Genomic DNA was extracted using the QIAGEN Genomic Kit. Next−generation sequencing (NGS) reads were generated on the Illumina HiSeq 4,000 platform, and third−generation sequencing was performed on the CCS sequencer (HiFi) with an average read length of over 50 kb. All sequencing steps were carried out by Beijing Biomarker Biotechnology Co. (Beijing, China) (**Supplementary Table 1**).

### Sequencing, genome assembly and annotation

The *de novo* assembly of NGS reads was performed using the default settings of GetOrganelle v1.7.5.3 (Jin et al., 2020), which successfully extracted the plastome. To obtain a complete and highly accurate mitogenome of *R. juparensis*, PMAT software was used to assemble PacBio HiFi reads (Bi et al., 2024). The final plastome and mitogenome were polished using NGS reads with Pilon v1.23 (Walker et al., 2014). GeSeq was used to annotate the plastome (Tillich et al., 2017), while the mitogenome was annotated using *Rhodiola crenulata* (OP312067.1), *Rhodiola sacra* (OP312070.1 and OP312071.1), and *Rhodiola wallichiana* (OP312069.1 and OP312068.1) as references. All tRNAs were predicted by tRNAscan−SE software (Chan and Lowe, 2019). Finally, the plastome and mitogenome were visualized using OGDRAW (Greiner et al., 2019).

### ENc-GC3s analysis

The effective number of codons (ENc) values reflect the degree of codon bias for the 20 amino acids in the ORF region of PCGs, with values ranging from 20 to 61. Values close to 20 indicate a preference for only one synonymous codon, while values close to 61 suggest that each synonymous codon is used equally. ENc plots (ENC−GC3s) are commonly used to assess codon usage patterns in genes. In this study, we used CodonW v1.4.4 software (Parvathy et al., 2022) for codon usage analysis and Geneious software (Kearse et al., 2012) for GC content analysis. The relationship between ENc and GC3s was visualized using R scripts (https://github.com/taotaoyuan/myscript). Predicted ENc values that lie on or above the expected curve indicate that codon usage is only influenced by G + C mutations. However, if natural selection or other factors are at play, the predicted ENc values will lie below the expected curve (Wright, 1990).

### Phylogenomic analysis

Due to the scarcity of mitogenome data, we were unable to use mitochondrial data to reconstruct the phylogenetic relationships of the subfamily Sedoideae Berger. Instead, we obtained 57 plastomes from NCBI to reconstruct the phylogenetic relationships of the subfamily Sedoideae Berger. These 57 plastomes comprised two *Sinocrassula* species, eight *Orostachys* species, seven *Hylotelephium* species, one *Rosularia* species, ten *Sedum* species, 28 *Rhodiola* species, and one outgroup *Batrachium* species. We extracted a total of 79 protein−coding genes (PCGs) using PhyloSuite (Zhang et al., 2020), aligned their CDS sequences with MAFFT v7.490 (Katoh et al., 2005), and removed poorly aligned regions using the ‘automated1’ parameter of trimAl (Capella-Gutiérrez et al., 2009). We constructed a concatenated matrix with FASconCAT−G (Kück and Longo, 2014) and used it to build a phylogenetic tree. We used MrBayes 3.2.6 (Ronquist et al., 2012) to infer the Bayesian inference (BI) tree, with the JC+I+G model selected by ModelFinder (Kalyaanamoorthy et al., 2017). Additionally, we inferred the maximum likelihood (ML) tree using IQ−TREE (Nguyen et al., 2015) with the GTR+F+I+G4 model and performed 5000 ultrafast bootstrap replicates.

The MCMCTree package of PAML v4.9j (Yang, 2007) was used to analyze divergence times. The fossil calibration points were mainly obtained from the Paleobiology Database (https://paleobiodb.org/) and the TimeTree5 website (http://www.timetree.org/). Two fossil calibration points were selected: *Ranunculus bungei* (126.0-132.4 Mya) and *Sedum lineare*−*Sedum sarmentosum* (0.420 Mya). Finally, the ChiPlot online website (Xie et al., 2023) was used to visualize the results.

### Identification of homologous sequences and transposable elements

We used Gepard software (Krumsiek et al., 2007) to identify possible structural rearrangements by aligning the plastomes of *R. juparensis*, *R. sacra*, and *Rhodiola tangutica*. The collinearity regions between the plastome and mitogenome of *R. juparensis* were identified using TBtools (Chen et al., 2020) with a matching rate of ≥ 80% and an E−value of ≤ 1e−5. The collinearity regions were visualized using the RIdeogram R package (Hao et al., 2020) with default parameters. The repetitive sequences within the plastome and mitogenome were identified and visualized by TBtools with an E−value ≤ 1e−5. TEs in the organelle genomes were identified using the CENSOR web server (Kohany et al., 2006) with “Viridiplantae” as a reference sequence.

### Estimation of nucleotide substitution rate

To compare the nucleotide substitution rate (NSR) of mitogenomes among the five *Rhodiola* species, we annotated 20 complete protein−coding genes (PCGs) from the draft mitogenome of *H. erythrostictum* and used these as a reference to calculate the NSR of PCGs in the five *Rhodiola* mitogenomes. We extracted 72 shared plastome PCGs and 16 mitogenome PCGs using PhyloSuite (Zhang et al., 2020) and calculated the synonymous (dS) and nonsynonymous (dN) substitution rates using KaKs_Calculator (Zhang, 2022) with the yn00 model. To estimate selection pressure resulting from the plateau environment, the five *Rhodiola* species were tagged as the foreground branch, and the Codeml package in PAML v4.9j (Yang, 2007) was used to identify potential PSGs. The tree structure is: “((*Rhodiola wallichiana*, (*Rhodiola crenulata*, ((*Rhodiola tangutica*, *Rhodiola juparensis*), *Rhodiola sacra*)))$1, (*Sedum plumbizincicola*, *Hylotelephium erythrostictum*));”. We initially performed branch-site likelihood ratio tests (LRTs) for each of the 13 protein-coding mitochondrial genes. Examination of the resulting LRT p-values revealed a marked skew toward values close to 1 (many > 0.9), which is consistent with the behavior previously documented for branch-site model analyses (Potter et al., 2021). Under such a p-value distribution, conventional multiple-testing corrections (e.g., Bonferroni, Benjamini-Hochberg FDR) are inappropriate because they assume a uniform distribution under the null hypothesis, applying them would be overly conservative and would substantially increase false negative rates. Therefore, instead of standard FDR correction, we followed the *post hoc* filtering strategy recommended by Potter et al. (2021) to control false positives while maintaining reasonable power. Specifically, we retained only genes satisfying: (1) uncorrected LRT p < 0.05 and at least one BEB site with posterior probability > 0.5 belonging to site class 2a or 2b, and (2) median interval between such BEB sites ≥ 10 amino acids.

### RNA editing analysis

To identify RNA editing sites in the plastome and mitogenome of *R. juparensis*, RNA sequencing reads were first quality−filtered and then mapped to the plastome and mitogenome references using HISAT2 (Zhang et al., 2021). Putative editing sites were identified by visually inspecting mapped reads in Geneious (Kearse et al., 2012). Stringent filtering criteria were applied to minimize false positives arising from sequencing errors and alignment artifacts. Specifically, an editing site was retained only if: (1) at least 5 % of the RNA reads covering the position supported the edited base, a threshold commonly adopted in plant RNA editing studies (Shi et al., 2016; Chen et al., 2017); (2) the total read depth at that position was sufficient to support this proportion-accordingly, positions with < 2.5 % of reads supporting the edited base were excluded to ensure statistical reliability; (3) the RNA editing efficiency (proportion of reads carrying the edited base) exceeded 20 %, as this cutoff filters out low−level “leaky” editing and background noise (Chen et al., 2017). Additionally, to ensure high−confidence identification, a minimum of two independent sequencing reads supporting the edited base was required. Our filtering strategy is consistent with recent RNA editing analyses in *Oryza sativa* and *Arabidopsis thaliana* (Liu et al., 2023). After filtering, the Pfam database (Mistry et al., 2021) was used to annotate proteins containing the pentatricopeptide repeat (PPR) domain in the unpublished *R. juparensis* genome.

## Results

### Characterization of the organelle genomes

**Figure 1:**
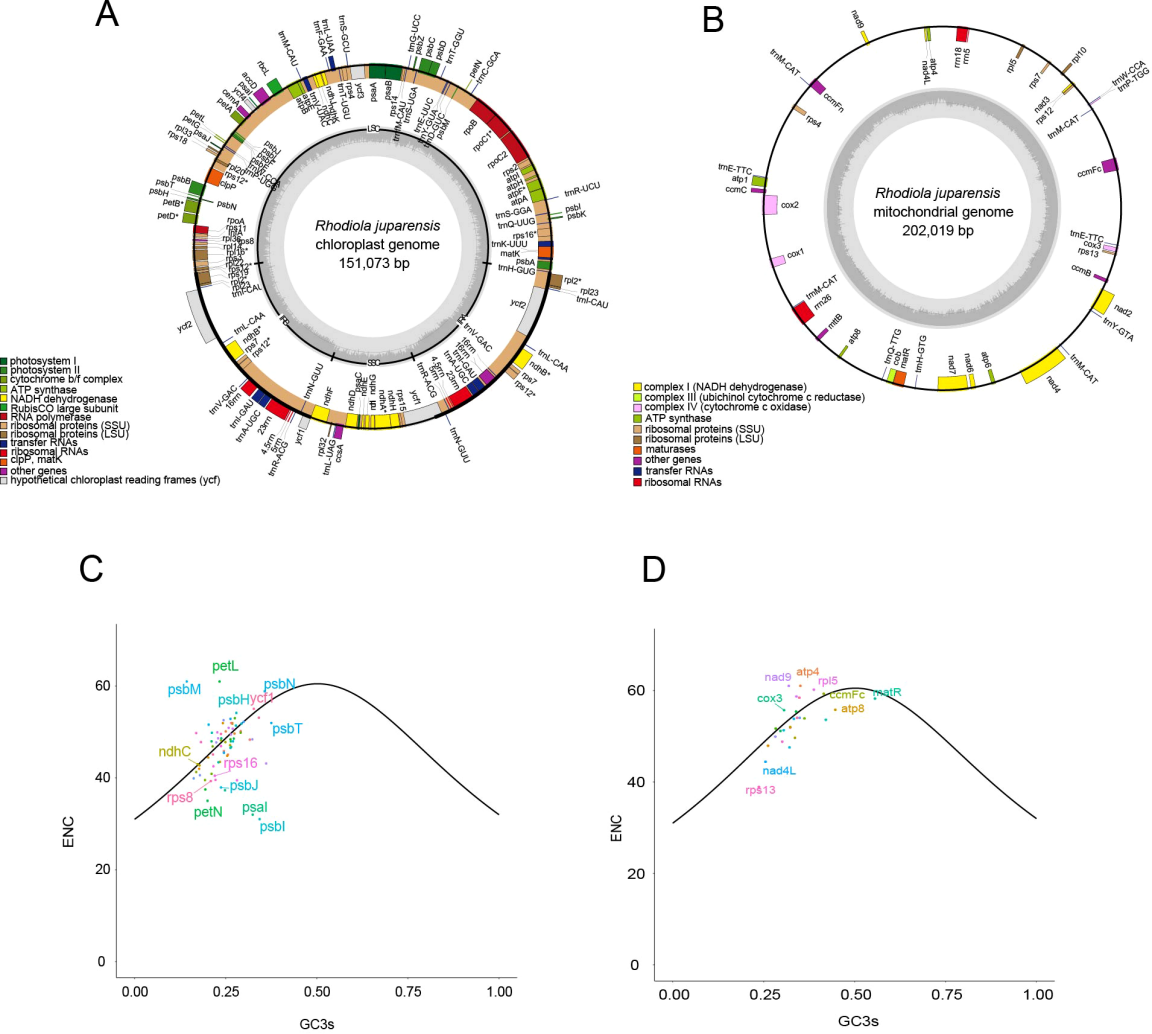
Genome maps and ENc plotted against GC3s of the organelle genomes. **(A)** Genome map of the plastome. **(B)** Genome map of the mitogenome. **(C)** ENc plot of the plastome. **(D)** ENc plot of the mitogenome. The outer circle shows gene locations, and the inner circle shows repeat sequence information. The links on the innermost circles represent repeats identified by BLASTn.

The plastome of *R. juparensis* exhibited a typical quadripartite structure with a total length of 151,793 bp (**Figure 1A**). It contained 128 unique genes, including 81 PCGs, 36 tRNA genes, and 8 rRNA genes. Among the PCGs, 40 were involved in photosynthesis and 58 in self-replication (**Table 1**). The mitogenome of *R. juparensis* assembled into a single circular chromosome of 202,019 bp, harboring 41 genes (**Figure 1B**), comprising 27 PCGs, 11 tRNA genes, and 3 rRNA genes (**Table 2**). The effective number of codons (ENc) values for plastome PCGs ranged from 31 to 61 (**Figure 1C**), whereas those for mitogenome PCGs ranged from 38 to 61 (**Figure 1D**). Only two plastome PCGs exhibited strong codon bias (ENc < 35), while no mitogenome PCGs had ENc values below 35, suggesting a generally weak codon preference in the mitogenome. The plastome and mitogenome of *R. juparensis* contained numerous TE fragments, with total lengths of 14,744 bp and 10,761 bp, respectively (**Table 3**). Repeat sequence analysis revealed that all repeats in the plastome were shorter than 100 bp (ranging from 40 to 92 bp), excluding the two inverted repeat (IR) regions (**Figure 1A**, **Supplementary Table 2**). In contrast, the mitogenome contained 32 repeat fragments totaling 30,610 bp, with the longest repeat sequence reaching 5,082 bp (**Figure 1B**, **Supplementary Table 3**). Additionally, 45 simple sequence repeats (SSRs) were detected in the plastome, whereas 22 microsatellites were identified in the mitogenome (**Supplementary Table 4**).

**Table 1:** Gene composition in the plastome of *R. juparensis*.

Category for genes	Function	Gene list
Genes for photosynthesis	ATP synthase	*atpA, atpB, atpE, atpF, atpH, atpI*
	ATP-dependent protease proteolytic subunit	*clpP*
	Photosystem I	*psaA, psaB, psaC, psaI, psaJ*
	Photosystem II	*psbA, psbB, psbC, psbD, psbE, psbF, psbH, psbI, psbJ, psbK, psbL, psbM, psbN, psbT, psbZ*
	Cytochrome b/f complex	*petA, petB, petD, petG, petL, petN*
	Rubisco large subunit	*rbcL*
	NADH dehydrogenase	*ndhA, ndhB, ndhC, ndhD, ndhE, ndhF, ndhG, ndhH, ndhI, ndhJ, ndhK*
Self-replication	Ribosomal RNAs (rRNA)	*rrn4.5, rrn5, rrn16, rrn23*
	Transfer RNAs (tRNA)	*trnA-UGC,trnC-GCA,trnD-GUC,trnE-UUC,trnF-GAA,trnfM-CAU,trnG-UCC,trnH-GUG, trnI-CAU,trnI-GAU,trnK-UUU,trnL-CAA,trnL-UAA,trnL-UAG,trnM-CAU,trnN-GUU, trnP-UGG,trnQ-UUG,trnR-ACG,trnR-UCU,trnS-GCU,trnS-GGA,trnS-UGA, trnT-GGU,trnT-UGU,trnV-GAC,trnV-UAC,trnW-CCA,trnY-GUA*
	Large subunit of ribosomal proteins (LSU)	*rpl2, rpl14, rpl16, rpl23, rpl22, rpl36, rpl20, rpl32, rpl33*
	Small subunit of ribosomal proteins (SSU)	*rps2, rps3, rps4, rps7, rps8, rps11, rps14, rps15, rps16, rps18, rps19*
	RNA polymerase	*rpoA, rpoB, rpoC1, rpoC2*
	Translational initiation factor	*infA*
Other genes	Maturase	*matK*
	Envelope membrane protein	*cemA*
	Subunit of acetyl-CoA-carboxylase	*accD*
	Cytochrome complex assembly	*ccsA*
Genes of unknown funcation	Hypothetical chloroplast reading frames	*ycf1, ycf2, ycf3, ycf4*

**Table 2:** Gene composition in the mitogenome of *R. juparensis*.

Group of genes	Name of genes
Transport membrane protein	*atp1, atp4, atp6, ATP8*
Cytochrome c biogenesis	*ccmB, ccmC, ccmFc, ccmFn*
Ubichinol cytochrome c reductase	*Cob*
Cytochrome c oxidase	*cox1, cox2, cox3*
Maturases	*matR*
Transport membrane protein	*mttB*
NADH dehydrogenase	*nad2, nad3, nad4, nad4L, nad6, nad7, nad9*
Large subunit of ribosome	*rpl5, rpl10*
Small subunit of ribosome	*rps4, rps7, rps12, rps13*
Ribosomal RNAs	*rrn5, rrn18, rrn26*
Transfer RNAs	*trnE-TTC, trnH-GTG, trnM-CAT, trnP-TGG, trnQ-TTG, trnW-CCA, trnY-GTA*

**Table 3:** Comparation of TEs in *R. juparensis* organelle genomes.

Type	Plastome		Mitogenome	
	Fragments	Length	Fragments	Length
DNA transposon	76	9070	42	3132
EnSpm/CACTA	14	1310	9	582
Harbinger	1	51	/	/
Helitron	24	3209	7	477
Mariner/Tc1	/	/	5	295
MuDR	25	3607	14	1357
hAT	12	893	6	353
LTR Retrotransposon	54	5144	53	6975
Copia	31	3623	31	3870
Gypsy	22	1418	22	3105
Non-LTR Retrotransposon	6	414	8	338
L1	5	359	8	338
SINE	1	55	/	/
SINE2/tRNA	1	55	/	/
Total	138	14744	205	10761
Ratio	/	9.71%	/	5.33%

The ratio was obtained by dividing the transposon sequence length by the genome length.

### Comparative plastome analysis

**Figure 2:**
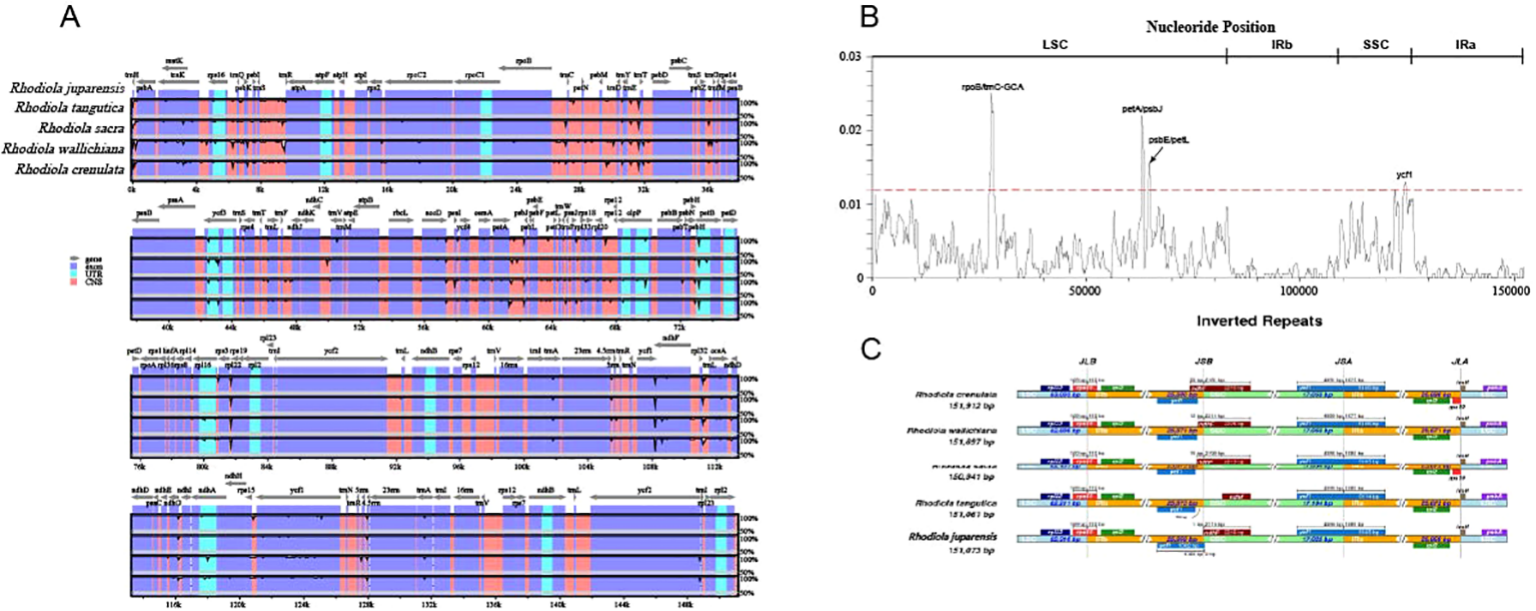
Syntenic analysis of plastomes among five *Rhodiola* species. **(A)** Comparison of the LSC, SSC, and IR region boundaries among five *Rhodiola* chloroplast genomes. **(B)** Nucleotide diversity (π) in the chloroplast genomes of five *Rhodiola* species. **(C)** Global alignment of chloroplast genomes of five *Rhodiola* species, with the *R. juparensis* genome as the reference.

To investigate sequence variability within the genus *Rhodiola*, we used the plastome of *R. juparensis* as a reference and compared the plastome sequences of five *Rhodiola* species using mVISTA software (**Figure 2A**). The analysis revealed intra−generic sequence variation, with highly divergent regions located mainly in intergenic regions (*rpoB-trnC-GCA* and *petA-psbJ*), as well as a variable region within the coding sequence of the *ycf1* gene (**Figure 2B**). Overall, the five plastomes showed high similarity in structure and gene order, indicating genome−wide evolutionary conservation of the plastome within the genus. We further compared the inverted repeat-small single−copy (IR−SSC) and inverted repeat-large single−copy (IR−LSC) boundaries among the five plastomes (**Figure 2C**). The genes *rpl19*, *ndhF*, *ycf1*, and *rps19* are located at the LSC/IRb, IRb/SSC, SSC/IRa, and IRa/LSC junctions, respectively. Specifically, *ndhF* is situated at the IRb/SSC boundary. Notably, in *R. crenulata*, *R. wallichiana*, and *R. tangutica*, *ndhF* has shifted into the IRb region, whereas in *R. juparensis*, *ycf1* has shifted into the SSC region. Despite these minor boundary shifts, the overall plastome structure remained consistent across the five *Rhodiola* species.

### Comparative mitogenome analysis

**Figure 3:**
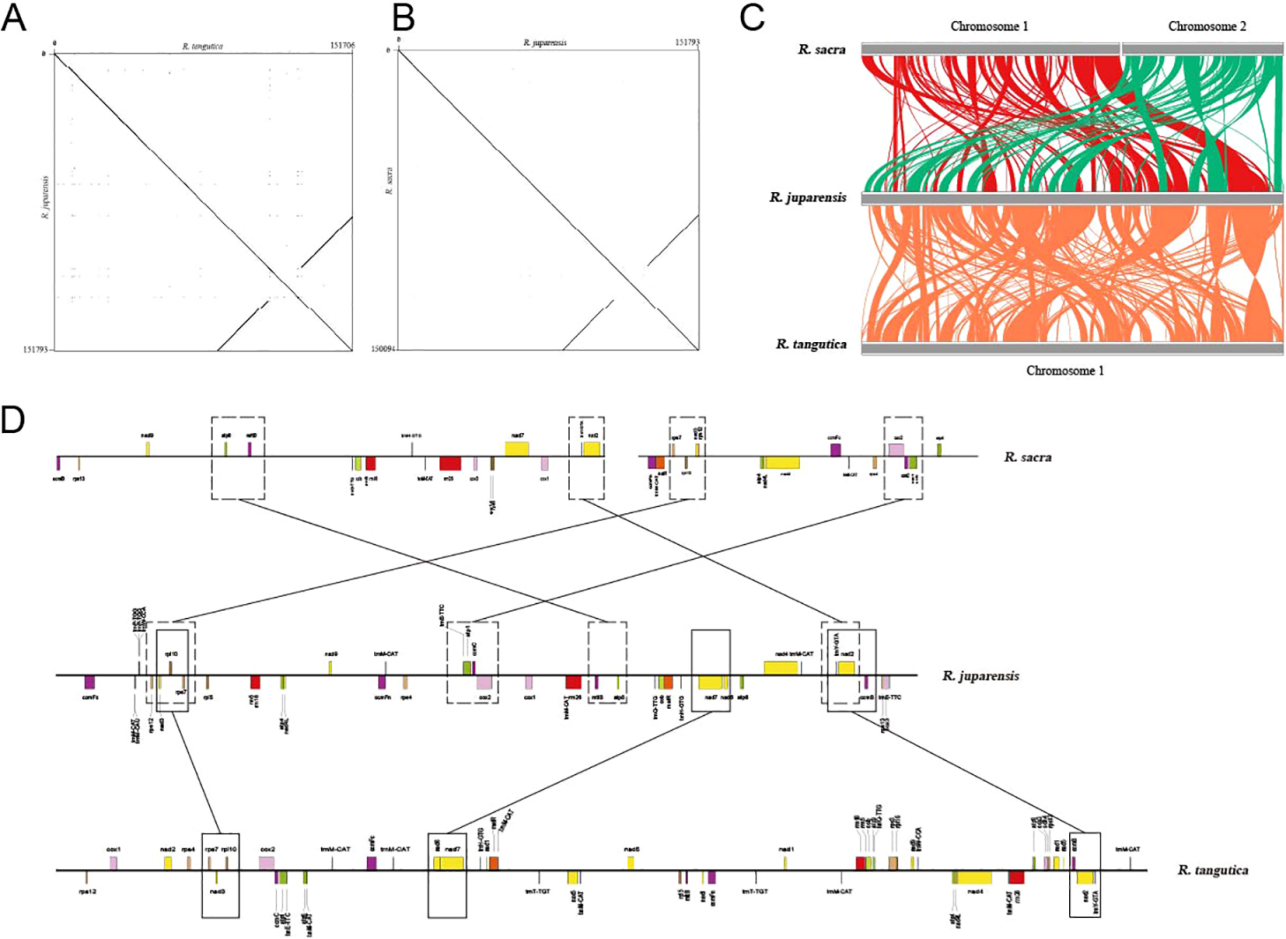
Syntenic analysis of organelle genomes among three *Rhodiola* species. **(A)** Syntenic regions between the *R. juparensis* and *R. tangutica* plastomes. **(B)** Syntenic regions between the *R. juparensis* and *R. sacra* plastomes. **(C)** Syntenic regions of mitogenomes among three *Rhodiola* species. **(D)** Conserved gene blocks among the mitogenomes of three *Rhodiola* species.

**Figure 4:**
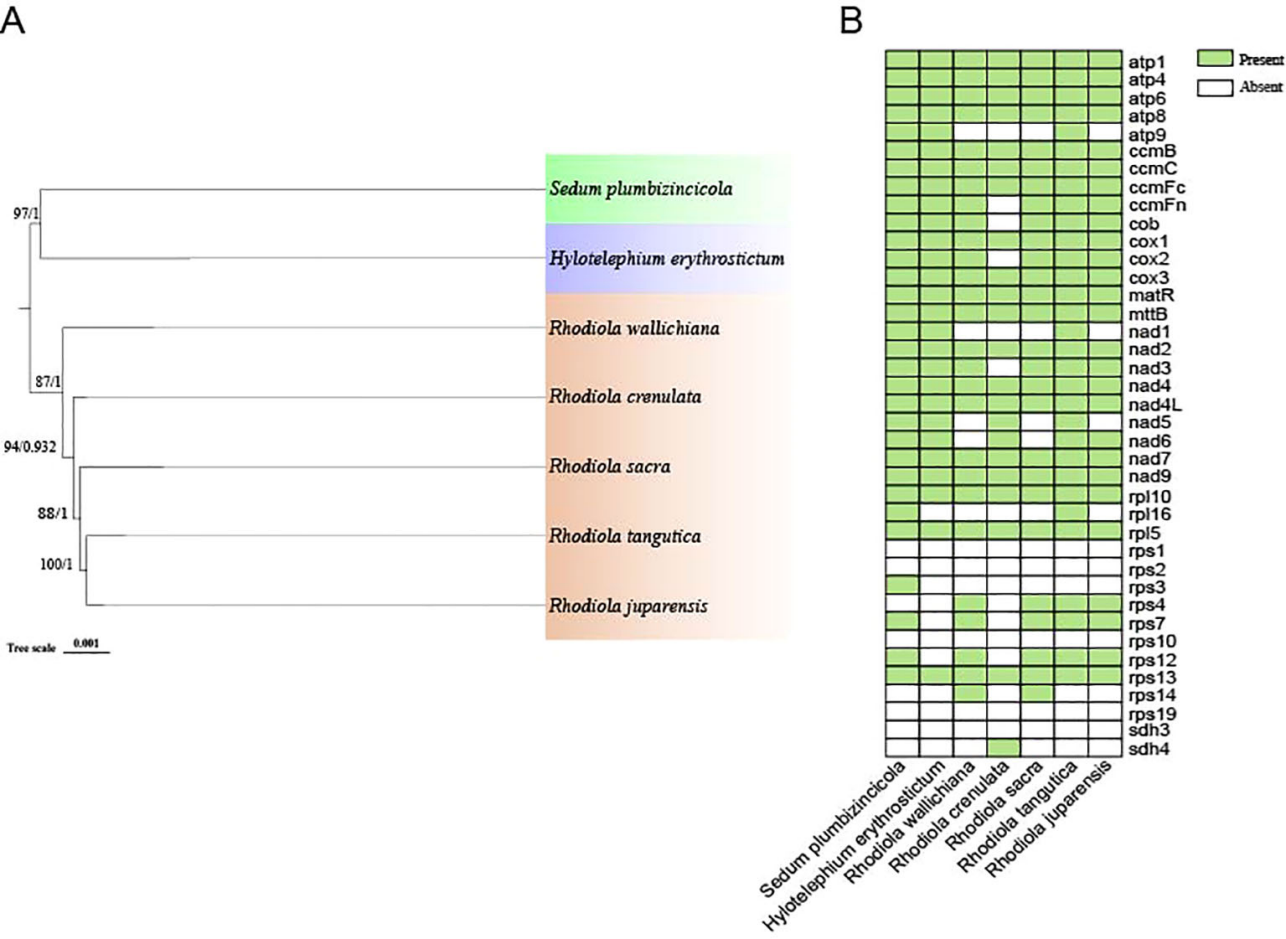
Mitogenome-based phylogeny and gene gain/loss. **(A)** Phylogenetic tree of *Rhodiola* species based on 79 shared PCGs from plastomes. Numbers below the branches represent ML bootstrap proportions/BI posterior probabilities. **(B)** Comparison of mitochondrial PCGs among Crassulaceae species.

Collinearity block analysis is commonly used to determine the evolutionary relationships among closely related species at the genome level. Therefore, we performed collinearity block analysis to explore the structural differences in the mitogenomes of *Rhodiola* species. Phylogenetic analysis results confirmed that *R. juparensis* was closely related to *R. sacra* and *R. tangutica.* Thus, we detected the homologous regions among the three organelle genomes. The plastomes of *R. juparensis*, *R. sacra*, and *R. tangutica* showed a high degree of collinearity (**Figure 3A**). On the other hand, we identified 165 collinearity blocks between *R. juparensis* and *R. tangutica* and 109 collinearity blocks between *R. juparensis* and *R. sacra* (**Supplementary Table 5**), indicating that the mitogenomes of the three *Rhodiola* species exhibited a more complex collinearity structure (**Figure 3B**). Due to the presence of a chromosomal fission in the *R. sacra* mitogenome forming two subgenomes, this appeared to result in reduced collinearity with *R. juparensis*. These collinearity blocks were dispersed throughout the genomes, suggesting that structural rearrangements have occurred in the mitogenomes of *Rhodiola* species. This phenomenon has also been observed in other taxonomic groups, such as *Populus simonii* and *Populus deltoides*, among others (Bi et al., 2022; Qu et al., 2022; Li et al., 2025). We also compared the positions of the orthologous genes of the three *Rhodiola* species and found that structural rearrangements disrupted gene clusters in the mitogenome (**Figure 3C**). However, some gene clusters were still preserved in *Rhodiola* species, including the *cox2*−*ccmC*−*atp1*−*trnE*−*TTC* and *rps7*−*rpl10*−*nad3*−*rps12* gene clusters shown in **Figure 3C**. In addition, we found a significant loss of mitogenome PCGs in the five *Rhodiola* species, including *rps1*, *rps2*, *rps3*, *rps10*, *rps19*, and *sdh3* (**Figure 4**). Previous studies have reported the presence of plastome gene residues in the mitogenome, implying significant sequence transfer between the two organelles (Zhang et al., 2024, 2025b, 2025a). To identify potential gene transfer fragments between the plastome and mitogenome, we conducted a search using BLASTN and obtained a total of 21 fragments (**Supplementary Figure 1**, **Supplementary Table 6**). The longest fragment was 2,918 bp, and the total length of these sequences was 15,959 bp. After annotation, we found that the mitogenome contained intact plastome−derived PCGs, including *matK* and *atpF*, referred to as mitochondrial plastid DNAs (MTPTs). However, we did not find any intact mitogenome−derived PCGs in the plastome. Additionally, we observed that several *tRNA* genes (*trnl*−*GAU* and *trnA*−*UGC*) were highly similar in sequence between the plastome and mitogenome. Collinearity block analysis is commonly used to infer evolutionary relationships among closely related species at the genome level. We therefore performed such analysis to investigate structural differences in the mitogenomes of *Rhodiola* species. Phylogenetic analysis confirmed that *R. juparensis* is closely related to *R. sacra* and *R. tangutica*, accordingly, we detected homologous regions among the three organellar genomes. The plastomes of *R. juparensis*, *R. sacra*, and *R. tangutica* showed a high degree of collinearity (**Figure 3A**). In contrast, we identified 165 collinearity blocks between *R. juparensis* and *R. tangutica* and 109 blocks between *R. juparensis* and *R. sacra* (**Supplementary Table 5**), indicating that the mitogenomes of these three species exhibit a more complex collinearity structure (**Figure 3B**). Notably, the mitogenome of *R. sacra* has undergone a chromosomal fission, resulting in two subgenomes, which likely accounts for the reduced collinearity with *R. juparensis*. These collinearity blocks are dispersed throughout the genomes, suggesting that structural rearrangements have occurred in the mitogenomes of *Rhodiola* species. Similar phenomena have been observed in other taxonomic groups, such as *Populus simonii* and *Populus deltoides* (Bi et al., 2022; Qu et al., 2022; Li et al., 2025). We further compared the positions of orthologous genes among the three *Rhodiola* species and found that structural rearrangements have disrupted gene clusters in the mitogenome (**Figure 3C**). Nevertheless, some gene clusters remain preserved, including the *cox2*-*ccmC*-*atp1*-*trnE*-*TTC* and *rps7*-*rpl10*-*nad3*-*rps12* clusters (**Figure 3C**). Additionally, we observed a substantial loss of mitogenome PCGs across the five *Rhodiola* species, including *rps1*, *rps2*, *rps3*, *rps10*, *rps19*, and *sdh3* (**Figure 4**). Previous studies have reported the presence of plastome-derived sequences in mitogenomes, indicating significant inter-organellar gene transfer (Zhang et al., 2024, 2025b, 2025a). To identify potential gene transfer fragments between the plastome and mitogenome, we performed BLASTN searches and recovered 21 fragments (**Supplementary Figure 1**, **Supplementary Table 6**). The longest fragment was 2,918 bp, with a total combined length of 15,959 bp. Annotation revealed that the mitogenome contains intact plastome-derived PCGs, including *matK* and *atpF*, representing mitochondrial plastid DNAs (MTPTs). However, no intact mitogenome-derived PCGs were found in the plastome. Furthermore, we observed that several *tRNA* genes (*trnI*-*GAU* and *trnA*-*UGC*) are highly similar in sequence between the two organellar genomes.

### Phylogenetic analysis and divergence time estimation

**Figure 5:**
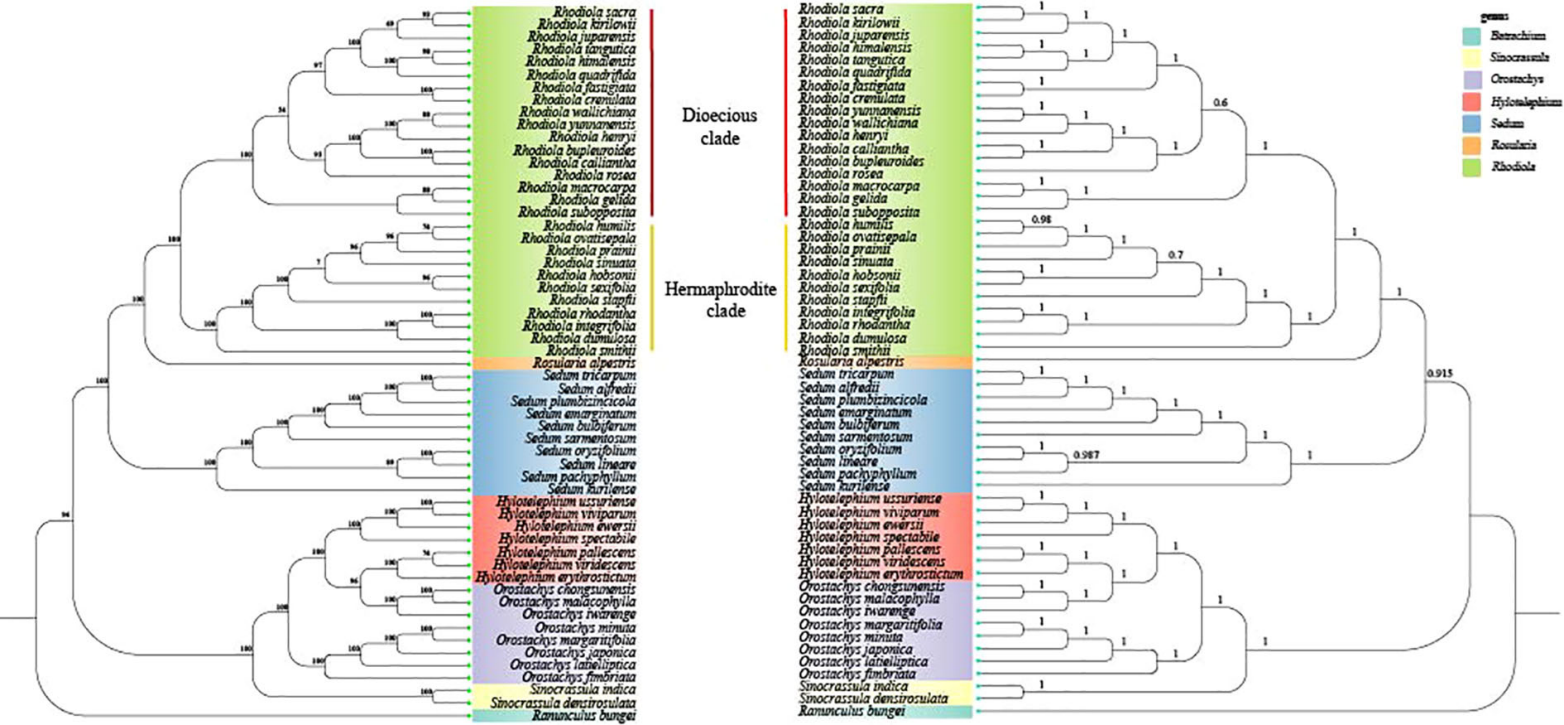
ML and BI trees constructed based on plastome data. The ML tree is shown on the left and the BI tree on the right. Numbers below the branches represent ML bootstrap proportions/BI posterior probabilities.

To determine the phylogenetic position of *R. juparensis* and to facilitate subsequent positive selection analysis, we reconstructed the phylogenetic relationships within the subfamily Sedoideae Berger using chloroplast genomic data. A total of 79 PCGs from 57 species were used to construct ML and BI trees (**Figure 5**). The two methods produced consistent topological relationships with high nodal support values. Overall, all Sedoideae species were divided into four clades, with all *Rhodiola* species forming a monophyletic group. At the intrageneric level, *Rhodiola* species were split into two branches, corresponding to dioecious and hermaphroditic individuals, respectively. Using *R. bungei* as an outgroup, we estimated divergence times. The results indicated that Sedoideae species underwent rapid radiative evolution approximately 7.15 million years ago (Mya). Within *Rhodiola*, *R. juparensis* was most closely related to *R. kirilowii* and *R. sacra* in our phylogenetic tree, with an estimated divergence time of approximately 0.43 Mya (**Supplementary Figure 2**).

### Analysis of NSR and positive selection in *Rhodiola* species

Given the predominant distribution of *Rhodiola* species on the QTP, we hypothesized that their organellar genes have experienced stronger selective pressure for high−altitude adaptation compared to species distributed at lower elevations. We selected a low−altitude outgroup species, *H. erythrostictum* (typically occurring at 400-1800 m above sea level), as a reference. Using the yn00 module in PAML v4.9j, we calculated pairwise NSRs for 55 shared plastome genes and 13 shared conserved mitogenome genes, including dN, dS, and the dN/dS ratio. Comparison of dN values among the five *Rhodiola* species revealed that *R. crenulata* and *R. juparensis* had significantly higher dN values in their plastome genes than the other three species (**Supplementary Figure 3**; **Supplementary Table 7**, **S8**), indicating a faster mutation rate in the plastomes of these two species. Additionally, *R. juparensis* and *R. tangutica* exhibited higher dS values in their plastomes. Notably, the relatively high dN and dS values observed in *R. juparensis* may suggest that this species has experienced stronger environmental selection pressure and is undergoing rapid evolution. We next analyzed dN and dS values for mitogenome genes across the five *Rhodiola* species (**Supplementary Figure 3**, **Supplementary Table 9**). *R. sacra* and *R. tangutica* showed higher dN values, whereas the dS values of mitogenome genes mirrored those of plastome genes, with *R. juparensis* and *R. tangutica* again displaying higher dS values (though not statistically significant). Collectively, these results indicate that the mutation rate of mitogenome genes in *Rhodiola* species is lower than that of plastome genes, particularly with respect to synonymous mutation rates (**Supplementary Figure 3**). We further performed Mann−Whitney U tests comparing dN and dS values between plastome PCGs and mitogenome PCGs. Both dS and dN were significantly higher in plastome PCGs than in mitogenome PCGs (dS p−value = 5.157e−07; dN p−value = 0.00623). These findings suggest that plastome genes tend to mutate faster than mitogenome genes, although some genes in the two organellar genomes mutate at similar rates.

Positive selection signals are often interpreted as imprints of environmental adaptation (Nielsen, 2005). To investigate this, we selected five *Rhodiola* species and two low−elevation species as outgroups for positive selection analysis based on available mitochondrial genomic data. A previous study on the *Rhodiola* plastome identified three PSGs, *ndhA*, *ndhH*, and *rpl16* (Zhao et al., 2020). The present study focused exclusively on mitogenomes and identified three PSGs (*atp8*, *ccmFc*, and *matR*) in the five high−altitude *Rhodiola* mitogenomes using the branch−site model **Table 4**.

**Table 4:** Positively selected genes and sites detected in the mitogenome of five *Rhodiola* .

Genome	Gene	Positive sites	LRT p-value
Mitogenome	*ATP8*	1,M,0.877;16,S,0.587;26,L,0.877;127,S,0.580;149,H,0.582;152,H,0.578	0.01125
	*ccmFc*	334,N,0.521; 366,S,0.835	0.03057
	*matR*	279,S,0.875	0.01269

In positive sites, integers represent the position of the site, letters represent the type of amino acid, and decimals represent the posterior probability.

### RNA editing and damage repair

**Figure 6:**
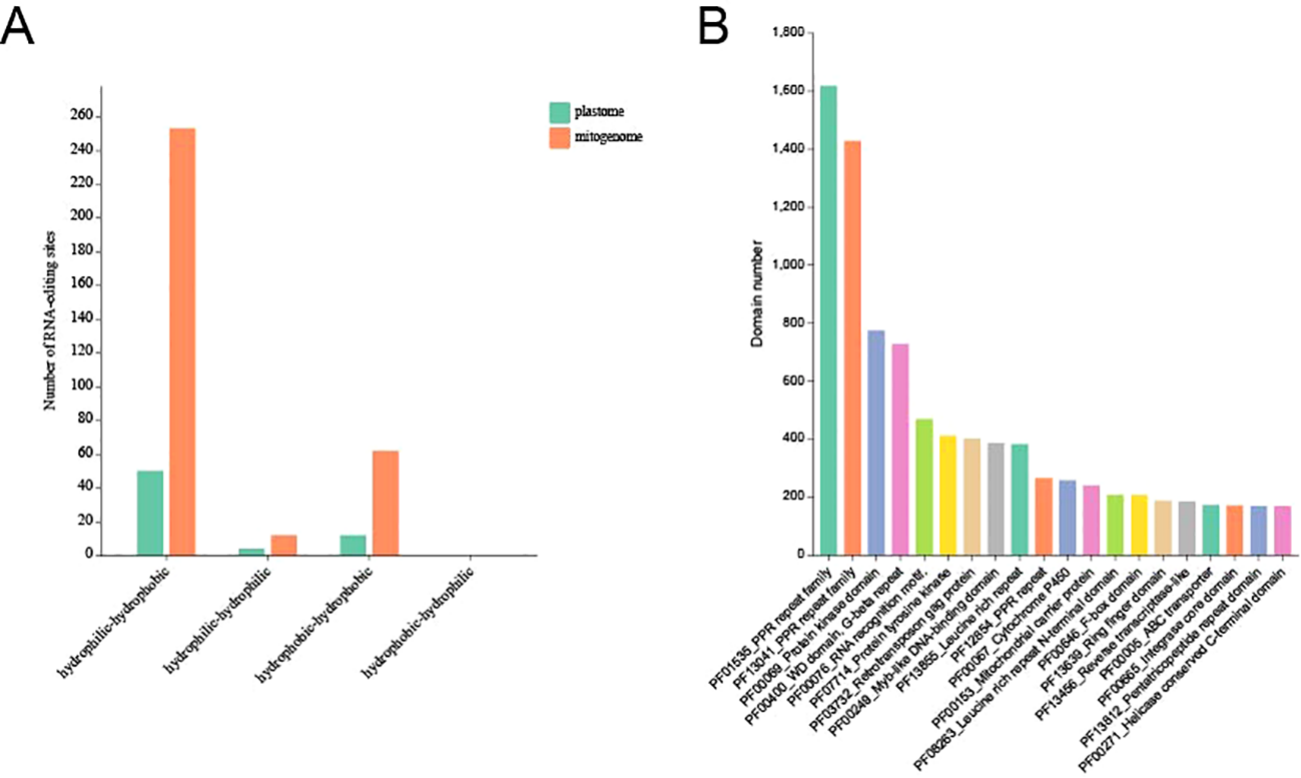
Effects of RNA editing on amino acid hydrophobicity and top nuclear genome domains. **(A)** Comparison of editing sites that lead to hydrophobicity/hydrophilicity changes in the resulting amino acid via nonsynonymous RNA editing. **(B)** Distribution of the top 20 domains in the nuclear genome.

After manual inspection, we identified 80 RNA editing sites in the plastome (**Supplementary Table 10**) and 343 RNA editing sites in the mitogenome (**Supplementary Table 11**), indicating that the mitogenome contains more editing sites. Within the plastome, 35 PCGs harbored editing sites, with 87.5% of the edits occurring at codon positions 1 and 2. Furthermore, 66 edits (82.5%) resulted in nonsynonymous amino acid changes, and 82.5% of the edits were C−to−U conversions. In the mitogenome, 27 PCGs contained editing sites, with 95.7% of the edits located at codon positions 1 and 2. Additionally, 327 edits (95.37%) led to nonsynonymous changes, and 72.9% of the sites were C−to−U edits. The plastome gene *ndhB* exhibited the highest number of editing sites (22), followed by *rpoC2* (6 sites). Among mitogenome PCGs, *nad4* had the most editing sites (40), followed by *nad7* and *ccmFn*, each with more than 20 sites. We also assessed the impact of editing on the hydrophobicity of encoded amino acids. The vast majority of nonsynonymous editing sites in both the plastome (75.8%) and mitogenome (73.8%) converted codons encoding hydrophilic amino acids to those encoding hydrophobic amino acids. Notably, we did not observe any editing events that converted hydrophobic codons to hydrophilic codons (**Figure 6A**).

Because PPR proteins are known to be extensively involved in RNA editing of organellar genomes, we analyzed PPR protein domains in the unpublished genome data of *R. juparensis* using the Pfam database. The results revealed that the *R. juparensis* genome contains a large number of PPR proteins (**Figure 6B**), which may provide the molecular basis for the extensive RNA editing observed in its organellar genomes.

## Discussion

### Organelles genome features

The plastome of *R. juparensis* was consistent with previous studies on *Rhodiola* species in terms of size, structure, and gene order, indicating that plastomes within the genus are evolutionarily conserved (Zhao et al., 2020; Yu et al., 2023). However, comparative genomic analysis revealed greater sequence divergence in non−coding regions than in plastome PCGs, such as in the *rpoB−trnC*, *petA*−*psbJ*, and *psbE*−*petL* intergenic regions. Additionally, the coding region of *ycf1* also exhibited a high mutation hotspot. These newly identified hypervariable regions (both genic and intergenic) may serve as useful molecular markers for phylogeographic and population genetics studies within *Rhodiola*. In contrast, mitogenomes showed substantial variation in structure, gene content, and gene arrangement. For example, the mitogenome of *R. sacra* comprised two circular loops and had lost up to 12 genes. By comparison, the mitogenome and plastome of *R. juparensis* exhibited similar structures, both being single loops. Furthermore, most PCGs in both the plastome and mitogenome of *R. juparensis* fell below the expected ENc curve, suggesting that natural selection was the primary factor shaping codon usage preferences (Zhang et al., 2022). This was especially true for photosynthesis−related and respiration−related genes, which are subject to strong environmental selective pressures under the harsh conditions (e.g., intense UV radiation) of the QTP. Nevertheless, not all photosynthesis− and respiration−related genes had ENc values below the expected curve, indicating that mutations also played a minor role in shaping codon preferences. Collectively, these findings indicate that natural selection is the dominant force driving codon usage bias in *R. juparensis*, particularly for functionally important genes such as those involved in photosynthesis and respiration (Jia et al., 2015). We also observed that the plastome exhibited a faster NSR compared to the mitogenome. These distinct genetic features between the two organellar genomes may be attributed to their different genome repair mechanisms (Smith and Asmail, 2014). Overall, this study contributes to understanding organellar genome evolution in *Rhodiola* species and provides a foundation for future investigations into the genetic mechanisms shaping plastome and mitogenome structure and function in plants.

### Gene loss, intracellular gene transfer and structural rearrangement

Unlike animals, which typically possess a nearly constant number of mitochondrial genes, mitogenome gene loss is common in many land plant lineages. The number of mitogenome genes varies widely, ranging from 19 in *Viscum scurruloideum* to over 50 in *Marchantia polymorpha* (Mower, 2009; Skippington et al., 2015; Johnston and Williams, 2016). In fact, loss of mitogenome genes, especially ribosomal protein genes, was a relatively frequent and persistent phenomenon in angiosperms (Petersen et al., 2015; Zervas et al., 2019). Most of these gene losses likely occurred following functional transfer of the genes to the nucleus, although gene loss does not necessarily imply functional nuclear transfer (Adams et al., 2001; Adams and Palmer, 2003). For example, nearly all ribosomal protein genes have been lost from the *Zostera* mitogenome, yet only small fragments of these genes have been identified in the nucleus (Petersen et al., 2015). It is plausible that mitogenome genes have been replaced by homologous genes derived from the plastome or nuclear genome (Adams et al., 2002).

In this study, we observed extensive gene loss events (*rps1*, *rps2*, *rps3*, *rps10*, *rps19*, *sdh3*) across the five *Rhodiola* species. Further analysis of homologous sequences revealed the presence of several intact plastome−derived PCGs in the mitogenome, whereas no intact mitogenome−derived PCGs were found in the plastome. These results are consistent with previous studies suggesting prevalent plastome−to−mitogenome DNA transfer in *Rhodiola* species (Yu et al., 2023) and indicate that such transfers may contribute to mitogenome gene loss in this genus. The direction of gene transfer appears to be unidirectional, supporting the view that the plastome is more receptive to DNA transfer than the mitogenome (Gao et al., 2014; Smith and Asmail, 2014). Inter−organellar gene transfer is an important process in plant evolution and is essential for growth and development. For instance, Cheng et al. demonstrated that transfer of the *Arabidopsis* plastome gene *AtRNH1C* to the mitogenome contributes to the maintenance of mitochondrial R−loop homeostasis and genomic stability during embryonic development, highlighting the importance of inter−organellar gene transfer in plant development and environmental adaptation (Cheng et al., 2021).

In addition to frequent gene loss and transfer, structural rearrangements are another hallmark of plant mitogenomes, reflecting their rapid structural divergence. Environmental stressors such as intense light and UV radiation have been suggested to promote mitogenome rearrangements, along with nuclear gene variation (Shedge et al., 2010; Xu et al., 2011; Virdi et al., 2016). For example, Xu et al. found that chromosomal rearrangements in *MSH1* mutants can alter plastid properties, thereby protecting plants from intense light damage (Xu et al., 2011). Therefore, the chromosomal rearrangements observed in *Rhodiola* species in this study may contribute to their adaptation to the extreme environmental conditions of the QTP, including intense light and UV radiation. Nevertheless, this hypothesis requires further experimental validation.

### Phylogenetic analysis and divergence time estimation

Phylogenetic analyses revealed that the ML and BI trees displayed a consistent topology, supporting *Rhodiola* species as a monophyletic group. At the intrageneric level, all *Rhodiola* species were divided into two branches corresponding to dioecious and hermaphroditic individuals, respectively, a finding consistent with previous studies (Zhao et al., 2020; Yu et al., 2023). Notably, the dioecious branch contained three dioecious species (*R. stapfii*, *R. prainii*, and *R. integrifolia*), of which *R. integrifolia* was tentatively classified as a hybrid of *R. rosea* and *R. rhodantha* in a prior study (Hermsmeier et al., 2012). We therefore hypothesize that *R. stapfii* and *R. prainii* may also be hybrids. Although this hypothesis requires further validation, our results suggest that *Rhodiola* species are divided into two clades based on sexual dimorphism, which may have significant implications for understanding the evolutionary processes driving diversification in this group. However, our analysis included only 28 *Rhodiola* species, future investigations into the molecular mechanisms underlying *Rhodiola* diversification will require more comprehensive approaches, including the use of multiple molecular markers, morphological data, and expanded taxon sampling to resolve the phylogenetic puzzle of this genus.

In this study, we observed that Crassulaceae species underwent rapid divergence primarily around 7 Mya. A recent study based on Late Miocene global sea surface temperature reconstructions suggested that a decrease in atmospheric CO_2_ levels approximately 7 to 5.4 Mya led to a global cooling event (Late Miocene cooling) (Tzanova et al., 2015; Herbert et al., 2016; Zhang et al., 2025b). Concurrently, a study by Chen et al. demonstrated that the QTP and the Xining Basin experienced dramatic uplift during the Late Miocene, which accelerated aridification in the Asian interior (Chen et al., 2019). We therefore hypothesize that the combination of CO_2_ reduction and QTP uplift drove the rapid divergence of Crassulaceae species during the Late Miocene, around 7 Mya.

### Positive selection analyses

Using the *Rhodiola* lineage as the foreground, we identified three candidate PSGs, *atp8*, *ccmFc*, and *matR*, in the mitogenomes of five high−altitude *Rhodiola* species using the branch−site model. These genes may be involved in plant adaptation to the extreme environment of the QTP. The *matR* gene is the only maturase enzyme gene retained in angiosperm mitogenomes and is highly conserved across land plants. It is thought to participate in the splicing of group II introns in plant mitogenomes. Sultan et al. demonstrated that reduced expression of *matR* leads to splicing defects in many pre−mRNA transcripts, and complete deletion results in embryonic lethality. Additionally, MatR−mediated splicing is critical for the maturation of *NAD1*, which is essential for plant respiration and stress response (Schmitz-Linneweber et al., 2015). Given that hypoxic conditions and intense UV radiation on the QTP could be fatal for early embryonic development and respiration in *Rhodiola*, we hypothesize that *matR* plays a role in maintaining mitochondrial function under such harsh conditions. Accordingly, we propose that *matR* contributes to early embryonic development and stress responses in *Rhodiola*. The *ccmFc* gene plays an important role in the maturation of cytochromes in mitochondrial complex III (Giegé et al., 2008), which is essential for mitochondrial function, plant growth, and development. Previous studies have shown that loss of *ccmFc* expression results in defects in c-type cytochromes and complex III, leading to impaired seed development or plant growth (Francs-Small et al., 2012). In *Arabidopsis*, deficiency of mature cytochromes caused by *ccmFc* mutation can lead to embryonic lethality (Meyer et al., 2005). Therefore, we hypothesize that *ccmFc* contributes to the ability of *Rhodiola* species to tolerate high-altitude stress and supports embryonic development, growth, and stress responses.

In pairwise comparisons, the *atp8* gene exhibited a relatively high dN/dS ratio when calculated against the outgroup *H. erythrostictum*, suggesting accelerated evolution of this gene. This result is consistent with the PAML branch−site model, which identified *atp8* as a PSG along the *Rhodiola* lineage. The convergence of both methods, despite their different statistical frameworks and reference points, supports the inference that *atp8* may have undergone positive selection during the adaptation of *Rhodiola* species to high−altitude environments. The *atp8* gene plays an essential role in the assembly of the ATP synthase holoenzyme, which is responsible for ATP synthesis. Mutations in *atp8* can lead to various effects, including altered mitochondrial structure and loss of function (Yu et al., 2009, 2020). Wang et al. showed that single nucleotide polymorphism (SNP) variants of the *atp8* gene in Tibetan yaks may be positively associated with high−altitude adaptation (Wang et al., 2018). Specifically, ten SNP loci differences in *atp8* resulted in amino acid changes that ultimately altered the conserved structural domain of cytochrome b561, potentially enabling more efficient utilization of scarce oxygen. We hypothesize that increasing altitude, accompanied by declining oxygen and carbon dioxide levels, imposes increased selective pressure on the *atp8* gene.

### RNA editing and damage repair

RNA editing is essential for regulating gene expression, RNA splicing, and plant growth and development. Mitochondrial RNA editing has evolved in diverse eukaryotic organisms, including slime molds (Byrne and Gott, 2004), land plants, and dinoflagellates (Waller and Jackson, 2009). In contrast, plastid RNA editing has only been reported in land plants, peridinin dinoflagellates, and fucoxanthin dinoflagellates (Lin et al., 2002; Howe et al., 2008). Notably, the predominant type of RNA editing in both mitochondria and plastids is C−to−U conversion. In this study, we identified 80 and 343 RNA editing sites in the plastome and mitogenome, respectively, suggesting that the mutational spectrum of mitochondrial DNA is broader and more variable than that of chloroplast DNA (Lynch et al., 2006). Importantly, 87.5% of plastome RNA editing events occurred at the first two codon positions, compared to 95.7% in the mitogenome. These findings are consistent with previous reports (Grimes et al., 2014; Zheng et al., 2020) indicating that most RNA editing sites result in amino acid changes at the first two codon positions (Zhang et al., 2022). We also observed that RNA editing primarily converts hydrophilic amino acids to hydrophobic ones (73.8% in the mitogenome and 75.8% in the plastome). Hydrophobicity is considered a major driver of correct protein folding (Moelbert et al., 2004; Li et al., 2016). Therefore, extensive RNA editing that increases hydrophobic amino acid content may facilitate the translation of mRNAs into correctly folded polypeptides, which is the structural basis for proteins to perform specific functions (Yura and Go, 2008).

PPR proteins have been recognized as key factors mediating organellar RNA processing (Kotera et al., 2005). PPR proteins bind to specific cis−acting elements, and PPR−RNA complexes can recruit additional binding factors, such as organelle RNA recognition motif−containing (ORRM) proteins and multiple organellar RNA editing factor (MORF) proteins, to form a higher−order editosome. ORRM proteins have been shown to be involved in RNA editing in *Arabidopsis* and *Oryza sativa* and are required for mitochondrial RNA editing (Shi et al., 2016). A previous study demonstrated a positive correlation between plastid RNA editing abundance and the size of the PPR gene family. To explore this relationship in *R. juparensis*, we performed genome−wide statistical analysis of PPR protein domains using unpublished genomic data and found that the *R. juparensis* genome contains a large number of PPR domain−containing proteins. This finding suggests that the numerous RNA editing sites observed in the organelles of *R. juparensis* may be associated with its high PPR protein content.

PPR proteins have been widely implicated in plant responses to abiotic stresses, including cold, drought, salinity, and oxidative stress (Xiao et al., 2021). Given that *R. juparensis* inhabits the QTP, where it is exposed to prolonged periods of intense UV−B radiation and low temperatures, it is plausible that its abundant PPR proteins contribute to adaptation to these harsh conditions. Notably, RNA editing has been proposed as a post−transcriptional mechanism that can compensate for certain types of DNA mutations at the RNA level (Meng et al., 2024), and emerging evidence suggests a role for RNA editing in the DNA damage response (Fujii and Small, 2011; Xhemalçe et al., 2024). However, direct experimental evidence supporting a role for PPR proteins in mitigating UV−induced DNA damage through RNA editing is currently lacking. Therefore, while our findings raise an intriguing hypothesis, functional studies, such as transcriptomic profiling under UV stress or targeted knockout of candidate PPR genes, are needed to test whether and how PPR proteins contribute to UV tolerance in *Rhodiola* species. Nevertheless, the abundance and diversity of PPR proteins in *R. juparensis* may reflect its unique adaptive strategy to cope with the extreme environmental conditions of the QTP.

## Conclusion

Comparative genomic analysis between the plastome and mitogenome of *R. juparensis* revealed that the plastome is more conserved in terms of structure, gene number, and gene order but evolves at a faster rate. In contrast, the mitogenome exhibits more gene loss and chromosomal rearrangements but evolves at a slower rate. Furthermore, gene transfer between organelles appears to be unidirectional, from the plastome to the mitogenome, with no transfer events detected in the opposite direction. Phylogenetic analysis supported *Rhodiola* as a monophyletic group and showed that dioecious and hermaphroditic species cluster into two separate branches. Divergence time estimation indicated that a rapid diversification of Crassulaceae species occurred around 7.15 Mya, which may be attributed to the decrease in atmospheric CO_2_ levels and the dramatic uplift of the QTP during the Late Miocene. Positive selection analysis identified *ccmFc*, *atp8*, and *matR* as candidate PSGs that may play critical roles in regulating the adaptation of *Rhodiola* species to the extreme environment of the QTP. RNA editing analysis revealed more editing sites in the mitogenome than in the plastome, and extensive RNA editing led to an increase in hydrophobic amino acids. This may facilitate the translation of mRNAs into correctly folded polypeptides, ultimately enabling the formation of three−dimensional structures with specific functions. Additionally, we hypothesize that the large number of PPR proteins in *R. juparensis* could act as “repair” factors to mitigate DNA damage caused by UV radiation in the QTP environment, although this possibility requires direct experimental validation. Overall, this study provides valuable insights into the genomic and adaptive evolution of *Rhodiola* and offers one of the few available genomic datasets for this genus.

## Data Availability

The raw transcriptome sequence data reported in this paper have been deposited in the Genome Sequence Archive in National Genomics Data Center, China National Center for Bioinformation/Beijing Institute of Genomics, Chinese Academy of Sciences (GSA: CRA044448) that are publicly accessible at https://ngdc.cncb.ac.cn/gsa. The plastome and mitogenome sequences generated in this study were deposited in GenBank database under accession numbers OR188140 and OR188139.
