# Supplementary material for: Structural divergence and molecular adaptation of Rhodiola juparensis organelle genomes
Source: Front Plant Sci. 2026 Jul 8;17:1881546. doi: 10.3389/fpls.2026.1881546 (PMC13390064; doi:10.3389/fpls.2026.1881546)
Supplement: Supplementary file 2 [file DataSheet1.docx]

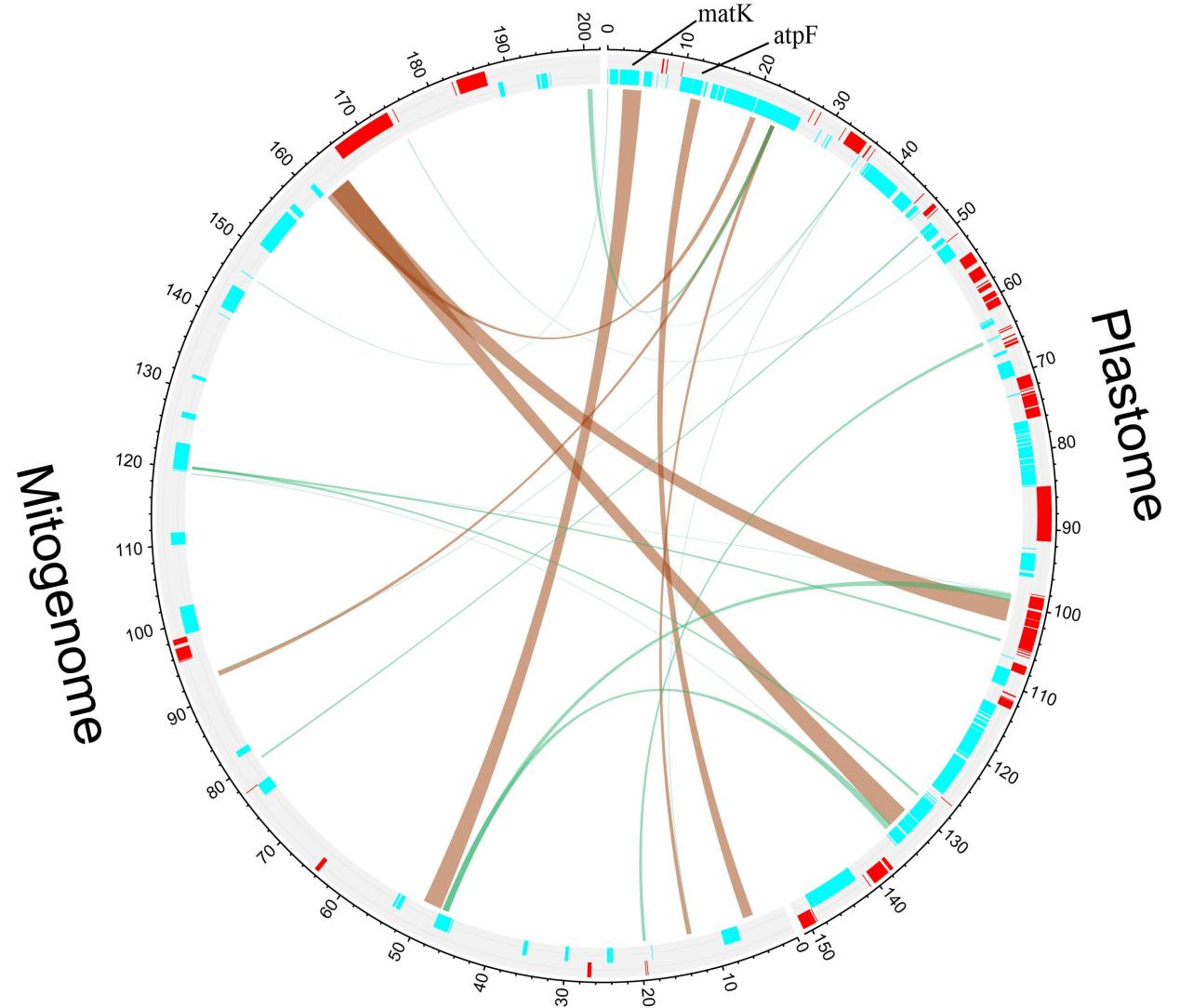


Figure S1 Homology sequences between plastome and mitogenome. On the circle plot, the red bars represent genes in the counter clockwise direction and the cyan bars represent genes in the clockwise direction. The shaded links represent identified homologous sequences. In homologous sequences, intact PCGs are highlighted with a broken line.


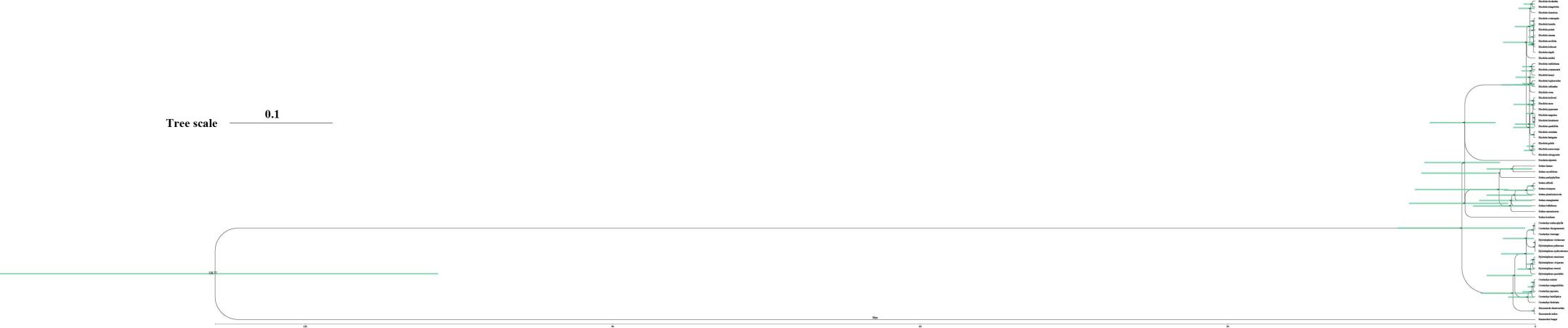


Figure S2 Chronogram showing divergence times among Crassulaceae plants with node age and 95% confidence intervals (green bars). The black numbers above the branches represent Divergence times.

**
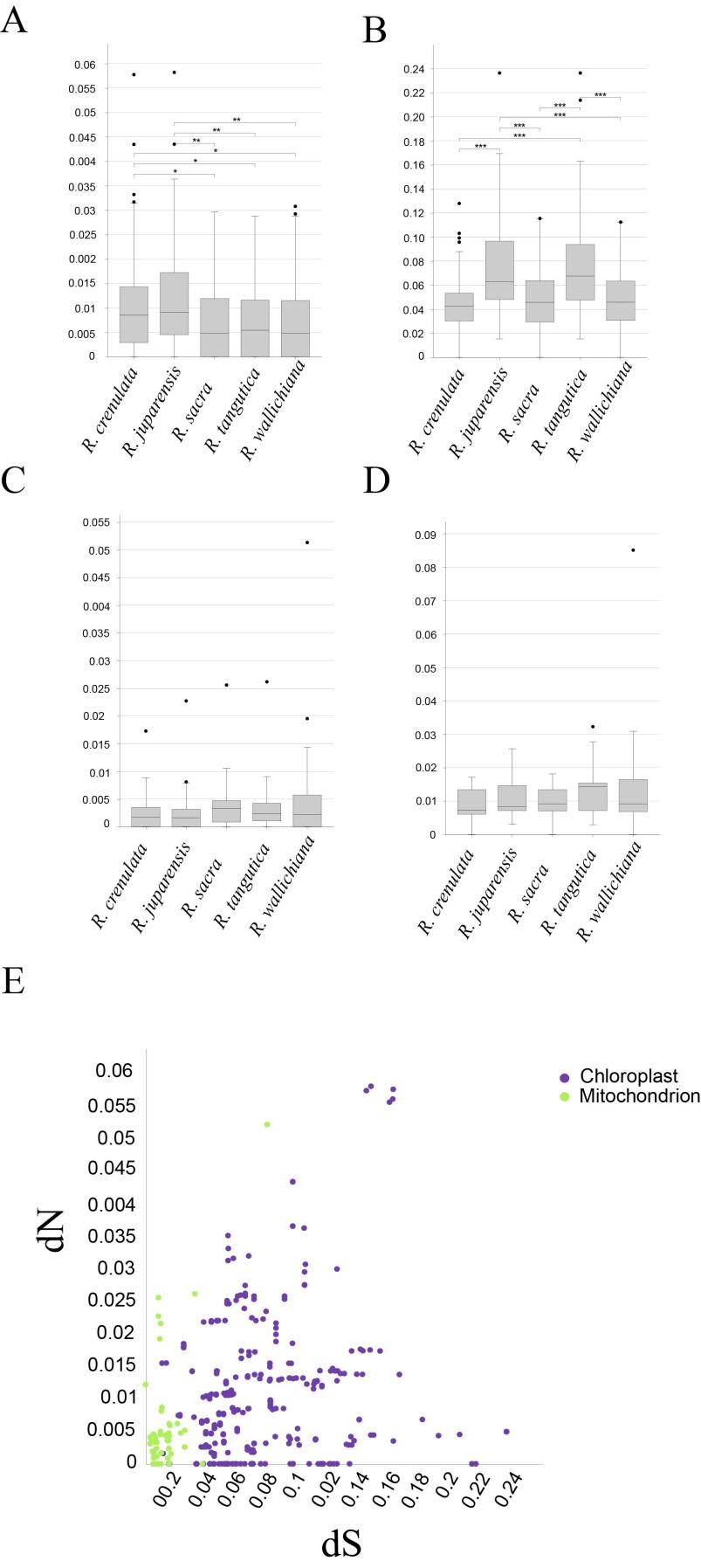
**

Figure S3 Variation in sequence divergence across species and organelles. (A) Comparison of dN values across three *Rhodiola* plastomes. (B) Comparison of dS values across three *Rhodiola* plastomes. (C) Comparison of dN values across three *Rhodiola* mitogenomes. (D) Comparison of dS values across three *Rhodiola* mitogenomes. (E) Comparison of dN and dS values across organelles.
